# Complete Genome Sequences of Two Bacteroides uniformis Bacteriophages

**DOI:** 10.1128/mra.00610-22

**Published:** 2022-09-19

**Authors:** Elsa M. Bean, Norma M. Morella, Neelendu Dey

**Affiliations:** a California Polytechnic State University, San Luis Obispo, California, USA; b Clinical Research Division, Fred Hutchinson Cancer Center, Seattle, Washington, USA; c Microbiome Research Initiative, Fred Hutchinson Cancer Center, Seattle, Washington, USA; d Department of Medicine, Division of Gastroenterology, University of Washington, Seattle, Washington, USA; DOE Joint Genome Institute

## Abstract

Here, we describe the isolation and genomic annotation of two novel siphovirus species of bacteriophages that infect Bacteroides uniformis: Bacteroides phage EMB1 and Bacteroides phage EMB2. EMB1 has a 34,204-bp genome with 48 coding sequences, and EMB2 has a 34,008-bp genome with 47 coding sequences.

## ANNOUNCEMENT

Bacteroides species are among the most abundant bacteria in the gut microbiome and have been linked to human health and disease (1). Bacteriophages (phages) are a crucial factor in shaping the structure and function of the human gut microbiome (2) and may be key components of future clinical interventions (3, 4). In this work, we sought to isolate and characterize phages for Bacteroides uniformis, which may have beneficial metabolic effects (5–7), as tools to study host-phage interactions in the human gut. Thus far, very few phages that infect B. uniformis have been isolated (8, 9).

Bacteroides phages EMB1 and EMB2 were isolated from filtered (0.22-μm pore size) primary effluent wastewater (collected 16 June 2021 from King County Wastewater Treatment Division’s West Point Treatment Plant, Seattle, WA). Host B. uniformis (strain ATCC 8492) cells were grown anaerobically at 37°C in a nutrient-rich bacterial growth medium (10). Phage enrichment and isolation were performed in growth medium supplemented with 100 μM taurocholic acid (catalog number T4009; Sigma-Aldrich), 100 μM glycocholic acid (catalog number G7132; Sigma-Aldrich), and 0.5% (wt/vol) mixed bile salts (catalog number 48305; Sigma-Aldrich). EMB1 and EMB2 were propagated on B. uniformis using liquid cultures in growth medium and the soft agar overlay method (11).

Genomic DNA was extracted using a phage DNA isolation kit (catalog number 46800; Norgen Biotek Corp.). Sequencing libraries were prepared using the Illumina DNA prep kit and IDT 10-bp unique dual indexes (UDI) and sequenced by the Microbial Genome Sequencing Center (MiGS, Pittsburgh, PA) on the Illumina NextSeq 2000 using 2 × 151-nt paired-end sequencing. Demultiplexing, quality control, and adapter trimming were performed by MiGS using bcl-convert version 3.9.3 (12). Read quality was assessed with FastQC version 0.11.9 (13), and quality filtering performed using BBMap version 38.92 (14). Fifty thousand paired forward and reverse reads (15) were randomly selected using Seqtq version 1.3-GCC-8.3.0 (16) and used for *de novo* assembly into contigs using MEGAHIT version 1.2.9 (17). Small contigs (213 to 2,412 bp) were determined to be residual bacterial genome sequencing, whereas phage genomes assembled into single contigs greater than 30,000 bp in size, had high coverage, and did not align to the B. uniformis genome. Quality trimmed reads were then mapped back onto each phage genome using BWA-MEM version 0.7.17-GCC-10.2.0 (18). Average genome coverage was determined using SAMtools Depth version 1.11-GCC-10.2.0 (19). Protein coding sequences (CDS) and tRNA genes were predicted and preliminarily annotated using Prokka version 1.14.5 (20). Putative functions were determined using BLASTp version 2.9.0 on the NCBI nonredundant protein sequence database (21) using a maximum expectation value of 0.001 (22). PhageTerm was used to predict the phage termini and packaging mechanism (23). The closest relatives to EMB1 and EMB2 were determined using nucleotide BLAST search (21) on the nucleotide collection (nr/nt) standard database. Intergenomic similarities of EMB1 and EMB2 to closest relatives and to each other were calculated using VIRIDIC Web (24). PhageTerm, Quast, and “GenBank Format to Five Column Format” were accessed through the Center for Phage Technology’s Galaxy and Web Apollo (https://cpt.tamu.edu/galaxy-pub) (25). Genome assembly results and accession numbers are summarized in Table 1.

**TABLE 1:** Phage genome assembly results and accession numbers

Phage	No. of:		Genome coverage (×)	GC content (%)	No. of:		Genome length (bp)	Packing mechanism	Termini	GenBank accession no.	SRA accession no.
	Sequencing reads	Filtered reads			CDS	tRNA genes					
EMB1	5,451,260	5,251,968	7,832	45.54	48	0	34,204	Unknown	Circularly permuted	ON721384	SRR19527454
EMB2	6,353,794	6,134,744	7,878	45.80	47	0	34,008	Headful (pac)	Circularly permuted	ON721385	SRR19527453

**FIG 1:**
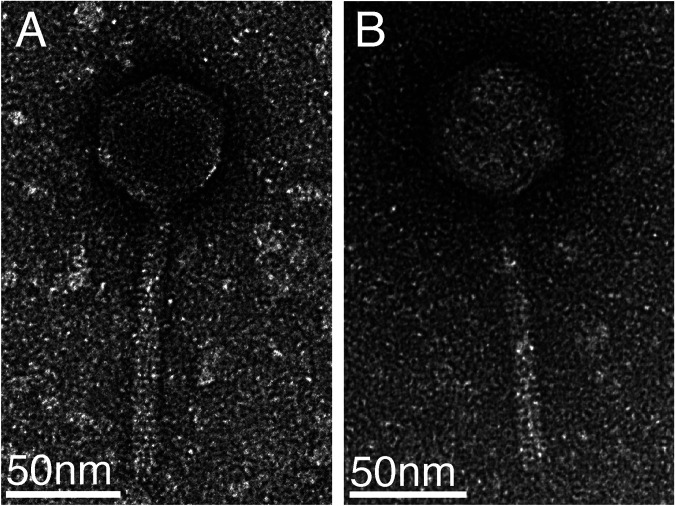
Transmission electron microscopy images of EMB1 (A) and EMB2 (B). Phage particles were fixed in 1/2 strength Karnovsky’s fixative overnight at 4°C and captured on Formvar/carbon-coated glow-discharged grids. Grids were negatively stained with 1% uranyl acetate and imaged on a ThermoFisher Talos L120c transmission electron microscope at an accelerating voltage of 120 kV. Six phage particles per isolate were measured using ImageJ (26) to determine approximate head and tail size.

EMB1 and EMB2 plaques are clear. Both phages have icosahedral heads (Fig. 1), and their head and tail sizes are consistent with *Siphoviridae* morphology (27). EMB1 is most closely related to phage ctND05 (GenBank accession number BK016558.1), with a nucleotide similarity of 86.6%, falling below the 95% average nucleotide identity (ANI) species cutoff (28); thus, EMB1 is a novel phage isolate. EMB2 is most closely related to phage ctND05 (GenBank accession number BK016558.1), with a nucleotide similarity of 87.9%. EMB1 and EMB2 have a nucleotide similarity of 90.8% to one another; therefore, EMB2 is a novel siphovirus as well.

### Data availability.

The GenBank accession numbers are ON721384 for EMB1 and ON721385 for EMB2. The SRA accession numbers are SRR19527454 for EMB1 and SRR19527453 for EMB2. Metadata are deposited under BioProject number PRJNA844182. BioSample accession numbers are SAMN28795846 for EMB1 and SAMN28795847 for EMB2.

## References

[1] Zafar H, Saier MH. 2021. Gut Bacteroides species in health and disease. Gut Microbes 13:1–20. doi:10.1080/19490976.2020.1848158. PMC7872030 33535896

[2] Shkoporov AN, Hill C. 2019. Bacteriophages of the human gut: the “known unknown” of the microbiome. Cell Host Microbe 25:195–209. doi:10.1016/j.chom.2019.01.017. 30763534

[3] Voorhees PJ, Cruz-Teran C, Edelstein J, Lai SK. 2020. Challenges & opportunities for phage-based in situ microbiome engineering in the gut. J Control Release 326:106–119. doi:10.1016/j.jconrel.2020.06.016. 32569705

[4] Pires DP, Costa AR, Pinto G, Meneses L, Azeredo J. 2020. Current challenges and future opportunities of phage therapy. FEMS Microbiol Rev 44:684–700. doi:10.1093/femsre/fuaa017. 32472938

[5] Fabersani E, Portune K, Campillo I, López-Almela I, la Paz SM, Romaní-Pérez M, Benítez-Páez A, Sanz Y. 2021. Bacteroides uniformis CECT 7771 alleviates inflammation within the gut-adipose tissue axis involving TLR5 signaling in obese mice. Sci Rep 11:11788. doi:10.1038/s41598-021-90888-y. 34083551 PMC8175583

[6] López-Almela I, Romaní-Pérez M, Bullich-Vilarrubias C, Benítez-Páez A, Gómez Del Pulgar EM, Francés R, Liebisch G, Sanz Y. 2021. Bacteroides uniformis combined with fiber amplifies metabolic and immune benefits in obese mice. Gut Microbes 13:1–20. doi:10.1080/19490976.2020.1865706. PMC8018257 33499721

[7] Gauffin Cano P, Santacruz A, Moya Á, Sanz Y. 2012. Bacteroides uniformis CECT 7771 ameliorates metabolic and immunological dysfunction in mice with high-fat-diet induced obesity. PLoS One 7:e41079. doi:10.1371/journal.pone.0041079. 22844426 PMC3406031

[8] Booth SJ, Van Tassell RL, Johnson JL, Wilkins TD. 1979. Bacteriophages of Bacteroides. Rev Infect Dis 1:325–336. doi:10.1093/clinids/1.2.325. 398578

[9] Hedžet S, Rupnik M, Accetto T. 2021. Novel Siphoviridae bacteriophages infecting Bacteroides uniformis contain diversity generating retroelement. Microorganisms 9:892. doi:10.3390/microorganisms9050892. 33919474 PMC8143477

[10] Dey N, Wagner VE, Blanton LV, Cheng J, Fontana L, Haque R, Ahmed T, Gordon JI. 2015. Regulators of gut motility revealed by a gnotobiotic model of diet-microbiome interactions related to travel. Cell 163:95–107. doi:10.1016/j.cell.2015.08.059. 26406373 PMC4583712

[11] Adams MH. 1959. Bacteriophages. Interscience Publishers, New York, NY. http://archive.org/details/bacteriophages00adam. Accessed 14 September 2021.

[12] Illumina. 2021. BCL Convert. https://support-docs.illumina.com/SW/BCL_Convert/Content/SW/FrontPages/BCL_Convert.htm. Accessed 27 May 2022.

[13] Andrews S. 2019. FastQC: a quality control tool for high throughput sequence data. Babraham Bioinformatics, Babraham Institute, Cambridge, UK. https://www.bioinformatics.babraham.ac.uk/projects/fastqc/.

[14] Bushnell B. 2014–2022. BBMap. https://sourceforge.net/projects/bbmap/. Accessed 10 August 2021.

[15] Russell DA. 2018. Sequencing, assembling, and finishing complete bacteriophage genomes, p 109–125. *In* Clokie MRJ, Kropinski AM, Lavigne R (ed), Bacteriophages. Springer, New York, NY. 10.1007/978-1-4939-7343-9_9 29134591

[16] Li H. 2022. Seqtk. https://github.com/lh3/seqtk. Accessed 27 May 2022.

[17] Li D, Liu C-M, Luo R, Sadakane K, Lam T-W. 2015. MEGAHIT: an ultra-fast single-node solution for large and complex metagenomics assembly via succinct de Bruijn graph. Bioinformatics 31:1674–1676. doi:10.1093/bioinformatics/btv033. 25609793

[18] Li H. 2013. Aligning sequence reads, clone sequences and assembly contigs with BWA-MEM. arXiv 1303.3997 [q-bio.GN]. https://arxiv.org/abs/1303.3997.

[19] Li H, Handsaker B, Wysoker A, Fennell T, Ruan J, Homer N, Marth G, Abecasis G, Durbin R, 1000 Genome Project Data Processing Subgroup. 2009. The Sequence Alignment/Map format and SAMtools. Bioinformatics 25:2078–2079. doi:10.1093/bioinformatics/btp352. 19505943 PMC2723002

[20] Seemann T. 2014. Prokka: rapid prokaryotic genome annotation. Bioinformatics 30:2068–2069. doi:10.1093/bioinformatics/btu153. 24642063

[21] NCBI Resource Coordinators. 2018. Database resources of the National Center for Biotechnology Information. Nucleic Acids Res 46:D8–D13. doi:10.1093/nar/gkx1095. 29140470 PMC5753372

[22] Pearson WR. 2013. An introduction to sequence similarity (“homology”) searching. Curr Protoc Bioinformatics 3:Chapter 3:Unit 3.1. doi:10.1002/0471250953.bi0301s42. PMC3820096 23749753

[23] Garneau JR, Depardieu F, Fortier L-C, Bikard D, Monot M. 2017. PhageTerm: a tool for fast and accurate determination of phage termini and packaging mechanism using next-generation sequencing data. Sci Rep 7:8292. doi:10.1038/s41598-017-07910-5. 28811656 PMC5557969

[24] Moraru C, Varsani A, Kropinski AM. 2020. VIRIDIC—a novel tool to calculate the intergenomic similarities of prokaryote-infecting viruses. Viruses 12:1268. doi:10.3390/v12111268. PMC7694805 33172115

[25] Ramsey J, Rasche H, Maughmer C, Criscione A, Mijalis E, Liu M, Hu JC, Young R, Gill JJ. 2020. Galaxy and Apollo as a biologist-friendly interface for high-quality cooperative phage genome annotation. PLoS Comput Biol 16:e1008214. doi:10.1371/journal.pcbi.1008214. 33137082 PMC7660901

[26] Schneider CA, Rasband WS, Eliceiri KW. 2012. NIH Image to ImageJ: 25 years of image analysis. Nat Methods 9:671–675. doi:10.1038/nmeth.2089. 22930834 PMC5554542

[27] Hendrix RW, Casjens SR, Lavigne. 2012. Family—Siphoviridae, p 86–98. *In* King AMQ, Adams MJ, Carstens EB, Lefkowitz EJ (ed), Virus taxonomy. Classification and nomenclature of viruses. Ninth report of the International Committee on Taxonomy of Viruses. Elsevier Academic Press, San Diego, CA.

[28] Adriaenssens E, Brister JR. 2017. How to name and classify your phage: an informal guide. Viruses 9:70. doi:10.3390/v9040070. PMC5408676 28368359

